# Maternal opioid use is reflected on leukocyte telomere length of male newborns

**DOI:** 10.1371/journal.pone.0261013

**Published:** 2021-12-17

**Authors:** Fatemeh Rahimi Mehdi Abad, Parvin Khalili, Fatemeh Jalali, Ali Pirsadeghi, Ali Esmaeili Nadimi, Azita Manshoori, Zahra Jalali

**Affiliations:** 1 Department of Clinical Biochemistry, School of Medicine, Rafsanjan University of Medical Sciences, Rafsanjan, Iran; 2 Social Determinants of Health Research Centre, Rafsanjan University of Medical Sciences, Rafsanjan, Iran; 3 Department of Epidemiology, School of Public Health, Iran University of Medical Sciences, Tehran, Iran; 4 Department of Pediatrics, School of Medicine, Rafsanjan University of Medical Sciences, Rafsanjan, Iran; 5 Non-Communicable Diseases Research Center, Rafsanjan University of Medical Sciences, Rafsanjan, Iran; 6 Molecular Medicine Research Center, Research Institute of Basic Medical Sciences, Rafsanjan University of Medical Sciences, Rafsanjan, Iran; 7 Department of Cardiology, School of Medicine, Rafsanjani University of Medical Sciences, Rafsanjan, Iran; 8 Department of Gynecology, School of Medicine, Rafsanjan University of Medical Sciences, Rafsanjan, Iran; Texas A&M University College Station, UNITED STATES

## Abstract

Opioid use accelerates normal aging in adults that raises a question on whether it may trans-generationally affect aging and aging biomarkers in the offspring of users as well? In the present research, we investigated the relative telomere length in umbilical cord blood of newborns born to opioid consuming mothers compared to normal controls. Telomere length shortening is a known biomarker of aging and aging related diseases. Its measure at birth or early in life is considered as a predictor of individual health in adulthood. Here, we performed a case-control study to investigate whether maternal opioid use affects newborns relative telomere length (RTL). 57 mother-newborn dyads were included in this study, 30 neonates with opioid using mothers (OM), and 27 with not-opioid using mothers (NOM)). RTL was measured in leukocyte cells genomic DNA using real-time PCR. The correlation of maternal opioid use with neonates telomer length was assessed using logistic regression analysis. The results displayed a significant association between odds ratio of long RTL and maternal opioid use when sensitivity analysis was performed by neonate sex; where the data indicates significantly increased odds ratio of long leukocyte RTL in association with maternal opioid use in male neonates only. Further work is necessary to assess this association in larger samples and test the potential underlying mechanisms for this observation.

## Introduction

Opioids are substances that bind to opioid receptors in different tissues such as brain and exert potent physiological effects, most importantly pain reduction and euphoria [1]. Today, the high rate of illicit opioid use in women is a critical public health concern, not only for women, but also for the risks it poses to their offspring, since opioids cross the placental and blood-brain barriers [2]. Pregnancy complications associated with prenatal opioid exposure include premature rupture of membranes, preeclampsia, spontaneous abortion, abruption placentae, and fetal death [3]. The prior studies on health issues for neonates born to opioid using mothers have mainly focused on the abstinence syndrome and the neurological impacts at infancy [4–6]. To the best of our knowledge, no study has addressed life-time and long-term consequences of prenatal opioid exposure.

There are several markers measured at birthtime that can help to predict future risk of chronic disease in adulthood. One of these markers is telomere length [7]. Telomeres are noncoding tandem repeats of DNA at the ends of chromosomes, essential to support genomic integrity and to protect DNA from degeneration or fusion with other chromosomes [7]. With each cell division, telomeres of somatic cells shorten until they reach a critical point that is damaging to the chromosomes, and consequently results in DNA damage response-mediated disruption of cell function and eventually leads to cellular senescence [8, 9].

Telomere length is considered to be correlated between somatic cells of various tissues, shortening with almost equal rates among different tissues [10]. Therefore, telomere length of blood cells is used in human studies as a peripheral surrogate for other tissues. In adults, there are reports of associations between shorter blood cell telomeres and development of chronic diseases such as cancer, cardiovascular disease, and type 2 diabetes [8, 9, 11], Alzheimer’s disease [12] and vascular dementia [13] as well as all-cause mortality [14]. Genetic and environmental factors regulate telomere length throughout the lifespan of each person, starting from in utero conditions [15–17]. Tracking telomere length from birth to adulthood, it was recently shown that the newborn telomere predicts later life telomere length [18], pointing to the importance of determining the initial state of newborn telomere and its significance for later life [19]. Maternal exposures in pregnancy are critical predictors of telomere length in offspring as a long-term trajectory determining the susceptibility of the individual to chronic diseases [20]. For example, previous studies in pregnancy associated psychosocial stress, gestational diabetes, metabolic syndrome, and tobacco exposure with altered telomere length in newborns [16, 21–32].

There are reports that have linked substance use of a person (i.e., cocaine, alcohol, heroin, and opioid) with acceleration of their normal aging, suggesting a role for accelerated telomere shortening (in one generation) [33–36]. However, the intergenerational impact of substance use on offspring aging hallmarks such as telomere length is not studied yet. In this regard, prior studies have focused on the features of the abstinence syndrome, anthropometric measures and neurological characters of neonates exposed to prenatal substance use at birthtime. However, similar research on the predictor markers of future health of offspring are warranted. Opioid use stimulates telomere shortening and age-related disease in adults [36–39], which raises a question how it may affect aging and aging biomarkers trans-generationally in the offspring of users? In the current research, we aimed to study telomere length in umbilical cord blood of newborns born to opioid consuming mothers compared to normal controls.

## Material and methods

### Participant recruitment, data collection and ethical approval

For this study, we recruited 67 healthy pregnant women aged 18–43 years who delivered neonates at Nick Nafs maternity hospital of Rafsanjan in year 2018. The eligibility criteria of mothers were as follows: A resident of Rafsanjan city, agreed to provide cord blood sample and completed the questionnaire interview, and completed and signed a written consent form. With the help of the maternity ward nurse and gynecologist, interview was performed and information on regular consumption of opioid by mothers was collected in the corresponding checklist. Details regarding the participants characteristics (neonate anthropometry, maternal pre-pregnancy weight, maternal weight before delivery, mother’s age, medical history and education, baby’s sex, and gestational age at delivery, delivery type) were obtained using the recorded checklists filled by primary health centers during pregnancy screening visits, including results of physical examination of pregnant women by the gynecologist in the health centers, as well as neonates characteristics recorded at birth-time after examination by medical doctors and maternity ward nurse). The study protocol was approved by the ethics committee of Rafsanjan University of Medical Sciences (ethics code: IR.RUMS.REC.1397.080), and all subjects gave informed consent.

### Study population and inclusion/exclusion criteria

After considering the following inclusion and exclusion criteria, 57 mother-newborn pairs were included in our analysis. In the test group (n = 30), mothers regularly using the following opioids: opium, sookhteh, shireh, and heroin, were included. Mothers who regularly used other drugs such as crack, methadone and amphetamine were excluded from our study. In the control group (n = 27), individuals without any history of any drug use were included. Neonatal umbilical cord blood was collected in 5 ml EDTA tubes from infants at birth. Our exclusion criteria for both groups were a history of diabetes, gestational diabetes and other chronic diseases such as hypertension and cardiac disease, and subjects with incomplete questionnaire information or with bad DNA quality.

### DNA extraction and telomere length measures

We extracted approximately 100 μg DNA from the umbilical cord blood and processed it for genomic DNA as per manufacturer’s instructions QIAamp DNA Mini Kit (Qiagen, DNeasy Blood and Tissue Kit). DNA quantity and purity was assessed by a Nanodrop and DNA integrity was assessed by agarose gel-electrophoresis.

Real-time polymerase chain reaction (PCR) was used to measure the relative telomere length in DNA samples extracted from umbilical cord blood leukocyte cells, based on the assay described by Cawton et al. [40]. This assay determines the ratio of telomeric repeat copy number (T) to a nuclear single copy gene (36B4d) (S) copy number (T/S ratio) in each given sample. Next, we estimated RTL as T/S relative to a reference DNA sample included in every PCR run (reference DNA used for standard curve plotting). Thus, RTL represents the factor by which a sample differs from a reference DNA in its ratio of telomere repeat copy number to single copy gene copy number and is proportional to the average telomere length. PCR runs were all performed in triplicates using Applied Biosystems step one plus Real-Time PCR machine and Jena bioscience qPCR Master Mix reagent. We used 20 ng DNA as a template for all reactions. RTL ratio were determined for each given sample from the quantitative PCR (qPCR) cycle threshold (ΔCt) values obtained.

The primer sequences (from 5’→3’) were: tel1)forward primer), GGTTTTTGAGGGTGAGGGTGAGGGTGAGGGTGAGGGT; tel2(revers primer), TCCCGACTATCCCTATCCCTATCCCTATCCCTATCCCTA; 36B4d (forward primer) CAGCAAGTGGGAAGGTGTAATCC; 36B4d(revers primer), CCCATTC-TATCATCAACGGGTACAA. The thermal cycling profile for the single copy gene and the telomere started with 1 min at 95°C for hot start activation followed by 30 cycles of 15-s denaturation at 95°C and annealing/extension at 61°C for 1 min.

### Sample size and power calculation

This study has been planned to include 52 participants, based on data from a previous study [37] in which the mean of telomere length was 1,0469 (SD = 1,783) in the control group and 6,901 (SD = 3,283) in the opioid addicted group, with a significance level of 0.05 (two-sided alpha of 0.025 to correct for two comparisons), and 90% power to detect of mean difference in the two groups. Thus, using the above estimates, the minimum sample size was estimated 13 people for each group in our study to measure telomere length of neonates with addicted mothers. Additionally, to determine the difference of the mean of telomere length in the two groups based on the sex of the infants, we doubled the estimated minimum sample size. Considering the problems with incomplete questionnaires or bad quality DNA samples, we recruited more participants (n = 67), from whom 57 subjects were included in our analysis, after excluding subjects with non-complete information or poor DNA quality.

### Statistical analysis

STATA 14 was used to perform all statistical analyses. For categorical values, chi-square test was used. In cases where >20% of the expected counts were less than 5, Fisher’s Exact test was performed. The data for the relative telomere length were subjected to the Shapiro–Wilk test to characterize normality. Because the samples did not have a normal distribution of relative telomere length (RTL), the Wilcoxon test was used to determine significant differences (p < 0.05). Additionally, logistic regression analyses were undertaken to assess the relationships between maternal regular opioid use and the outcome of interest, neonate RTL. Participants were categorized to two groups based on the median value of the RTL considering specific median value for male and female neonates. For our logistic regression analysis, variables included in the models were those that had a p value <0.1 in bivariate regressions (maternal education) (Hosmer and Lemeshow [41]), as well as other variables that had strongly been supported by literature to be associated with leukocyte telomere length in neonates (maternal age). To increase our power to detect differences (considering the small sample size, especially after stratification by sex of neonate), we decided to use two bivariate adjusted models each with one covariate included (mother’s age or education level), rather than including both covariates in one single model. Mothers age was used as a continuous parameter. Maternal education was categorized to three levels (1: illiterate or primary school, 2: middle and high school, 3: University degree). Neonate weight was categorized based on the median value for boys and girls, and neonates were thus divided to heavy and non-heavy categories. In order to test for the potential mediation (suppressor) effect of neonate weight on the association of RTL and maternal opioid consumption, a comparison was made between the odds ratio of logistic regression models assessing the association of RTL and OM, with (A, adjusted) and without (U, unadjusted) the neonate categorical weight parameter inclusion in the model (Table 3). The mediation proportion was estimated using the equation as follows: suppressed risk = ((OR_u_-OR_A_) /(OR_u_-1)) ×100. All *p*-values are two-sided, and *p*-values < 0.05 and 95% confidence intervals were considered as statistically significant.

## Results

**Fig 1:**
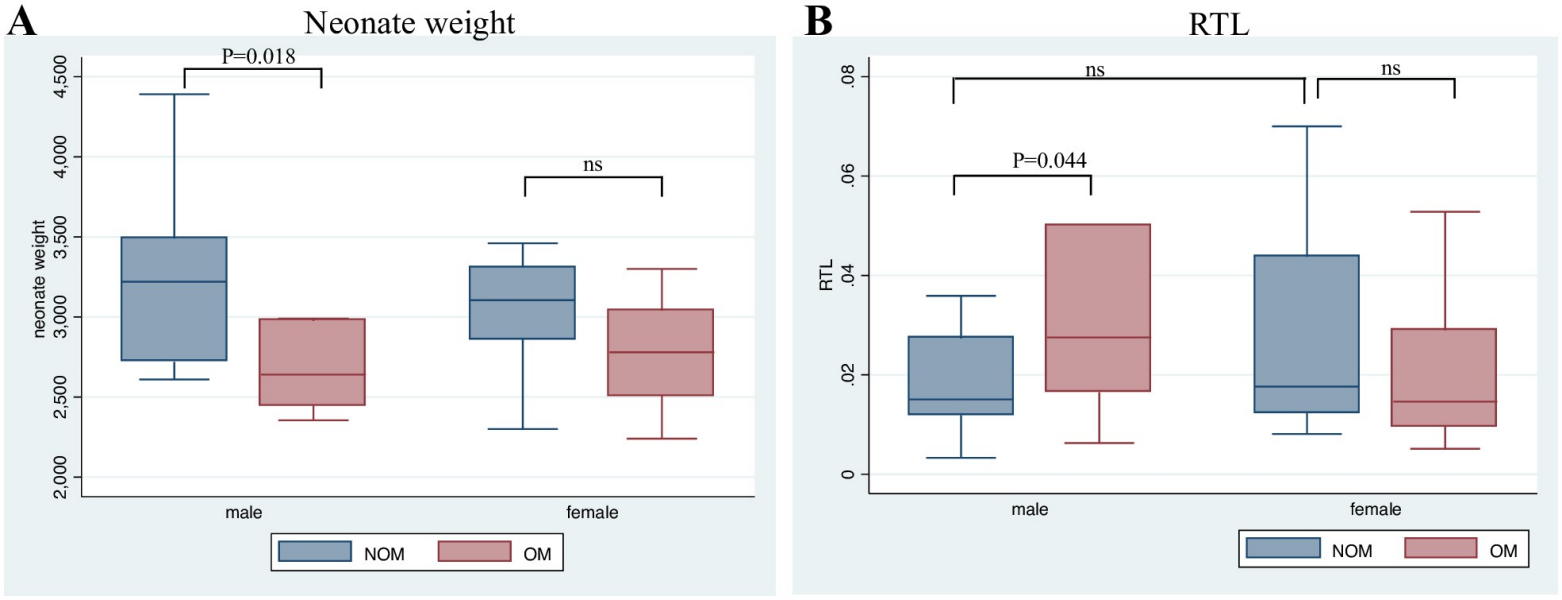
Neonate weight and relative telomere length (RTL) in OM and NOM offspring. Box plot diagram of neonate (A) weight, and (B) relative telomere length (RTL) in male and female offspring in OM and NOM groups. Box plot explanation: upper horizontal line of box, 75th percentile; lower horizontal line of box, 25th percentile; horizontal bar within box, median; upper horizontal bar outside box, maximum value except for outliers; lower horizontal bar outside box, minimum value except for outliers; p < 0.05 indicates statistical significance between groups based on Wilcoxon Rank Sum test. OM: opioid using mothers, NOM: Healthy controls (Not-opioid using mothers), ns: not significant.

A total of 57 mother-neonate dyads were included in the present study. Table 1 depicts the baseline information for the offspring and mothers i.e. neonate sex and anthropometric features (weight, height and head circumference), maternal age and weight before and after gestation in the two groups of neonates born to opioid user mothers (OM)(n = 30) and not-opioid user mothers (NOM, n = 27). There was no significant difference between OM and NOM regarding neonates’ sex. Neonate height was not significantly different in OM infants (OM vs. NOM height: 47.8 vs. 48.75 cm, p-value:0.08). Normal range of neonate weight is 2500–4000 grams, and a birthweight lower than 2500 is considered low birthweight (LBW). Among our participants, 7.41% of controls and 20% of OM subjects displayed LBW (Table 1). According to Shapiro–Wilk normality test, birthweight distribution was not normal in our sample. Running Wilcoxon test to compare birthweight in OM and NOM groups, the average value of neonate weight was significantly lower in OM infants (OM vs. NOM weight: 2828.5 vs. 3140.92 gram, p-value: 0.002). However, running the test separately for male and female offspring, the results only displayed significant difference between OM and NOM male (p = 0.018) and not female offspring (p = 0.0508) (Fig 1A).

**Table 1:** Baseline characteristics of the study population by maternal regular opioid use.

	NOM	OM	p-value	Total
Neonate Sex (%)			0.67	
Male	15(50.0)	15(50.0)		30(52.6)
female	12(44.4)	15(55.6)		27(47.3)
Preterm or term (%)			1.000*	
Preterm	1 (3.7)	2 (6.6)		3 (5.2)
Term	26 (96.3)	28 (93.3)		54 (94.7)
Low birthweight (birthweight <2500 grams) (%)			0.172*	
no	25 (92.59)	24 (80.0)		49 (85.96)
yes	2 (7.41)	6 (20.00)		8 (14.04)
Heavy baby (birthweight>median)			0.002*	
No	9 (33.33)	22 (73.33)		31 (54.39)
Yes	18 (66.67)	8 (26.67)		26 (45.61)
Maternal education level (%)			<0.001*	
Illiterate/primary school	1(3.85)	5 (16.67)		6(10.71)
Middle/high school	9 (34.62)	21 (70.00)		30 (53.57)
University (B.S and M.S)	16 (61.54)	4 (13.33)		20 (35.71)
Maternal smoking (%)			<0.001*	
Smoking	0 (0)	13 (43.33)		13 (22.81)
Non-smoking	27 (100)	17 (56.67)		44 (77.19)
RTL>median			0.066*	
Male offspring RTL >median (%)	5 (33.33)	11 (73.33)		16 (53.33)
Male offspring RTL <median (%)	10 (66.67)	4 (26.67)		14 (46.67)
RTL>median			0.69*	
Female offspring RTL > median (%)	8 (66.67)	8 (53.33)		16 (59.26)
Female offspring RTL < median (%)	4 (33.33)	7 (46.67)		11 (40.74)
**Mean ± SD**				
Neonate weight (g)	3140.92±488.91	2828.5±526.15	**0.002** ^#^	
Neonate height (cm)	48.75±2.30	47.8±1.78	0.08	
Male neonate RTL	1.05±0.24	2.05±0.46	**0.044** ^#^	
Female neonate RTL	1.79±0.63	1.09±0.25	0.40^#^	
Neonate head circumference (cm)	33.46±1.06	33.18±1.52	0.43	
Mother age (years)	31.74±6.45	31.27±6.12	0.78	
Mother weight before gestation (kg)	59.53±11.86	60.51±14.17	0.78	
Mother weight at gestation (kg)	74.68±13.65	70.28±18.39	0.31	

Data are given as mean±SD or absolute number n (percentage).

p-values for differences between categories were obtained using the t-test or Wilcoxon rank sum^#^ for continuous variables and using the χ2 or Fisher Exact* Test for categorical variables.

NOM: non-opioid using mother, OM: opioid using mother, RTL: relative telomere length.

The mean value for the maternal age (case vs. control: 31.2 vs. 31.7 years old, p-value: 0.78), maternal weight at gestation (case vs. control: 70.2 vs. 74.6 kg, p-value: 0.31), as well as weight before gestation (case vs. control: 60.5 vs. 59.5 kg, p-value: 0.78) were not significantly different between the two groups of study. Maternal education level was significantly different between the two groups with the majority of NOM (86%) had education level 1 and 2, while in OM group around 96% had education level 2 or 3 (p-value< 0.001) (Table 1). Smoking status was significantly different between the two groups where non-opioid using mothers were all non-smokers (Table 1, p-value<0.001). Among participants of our study, no one reported a history of alcohol use.

We applied the Wilcoxon rank sum test to compare the relative telomere length in male and female neonates born to opioid using mothers and normal neonates. The results showed significantly longer relative telomeres in male neonates with opioid consuming mothers (p = 0.044) (Fig 1B). In female offspring, Wilcoxon test did not confirm a significant difference in the relative telomere length (p = 0.40) (Fig 1B). Mean value for RTL was 1.05 and 1.79 for male and female neonates, respectively.

Next, we performed logistic regression analysis to estimate the correlation of maternal opioid use with neonates telomer length. RTL was categorized in two groups based on the median value estimated for male and female offspring separately. Table 1 depicts the data for the mean values of RTL and the proportion of newborns with RTL higher than median (considered as long telomere category for male and female newborns in NOM and OM groups). As shown in Table 1 without stratification for sex, the crude and the two adjusted regression models displayed that the odds ratio of having long RTL does not significantly associate with maternal opioid use. However, when sensitivity analysis was performed by stratification of data set by neonate sex, the data indicates significantly increased odds ratio of long RTL in association with maternal opioid use only in sons (Crude model OR: 5.5 (95% CI: 1.14–26.41), p-value:0.03; adjusted model 1 OR: 4.95 (95% CI: 10.1–24.31), p-value: 0.04; adjusted model 1 OR: 7.3 (95% CI: 1.05–51.81), p-value: 0.04) (Table 2). Due to the small sample size, and the consequent low statistical power of our analysis after stratification for sex, we could only control for one confounding parameter in each of the adjusted models. In adjusted model 1, we included maternal age as the confounder, due to its strong correlation with neonate telomere length as supported by literature [42–45], and in the second adjusted model we controlled for maternal education level, since it displayed a significant difference between the two groups of our study (NOM vs. OM) (please see Table 1).

**Table 2:** Estimated crude and adjusted odd ratios for telomeres longer than median (long RTL) in neonate, as predicted by maternal regular opioid use.

	Crude model		Adjusted Model 1		Adjusted Model 2		Adjusted Model 3	
Long RTL	OR(95% CI)	p-value	OR(95% CI)	p-value	OR(95% CI)	p-value	OR(95% CI)	p-value
**Maternal regular opioid use (OM)**	1.86 (0.64–5.4)	0.25	1.77 (0.61–5.16)	0.29*	1.93 (0.57–6.52)	0.28**	1.48 (0.43–5.10)	0.532***
**Maternal regular smoking**	2.05 (0.55–7.67)	0.28	1.91 (0.49–7.39)	0.346 *	2.14 (0.55–8.24)	0.269**	1.66 0.35–7.80)	0.517****
**Sensitivity analysis by sex**								
**Maternal regular opioid use (OM), Sons**	5.5 (1.14–26.41)	**0.03**	4.95 (10.1–24.31)	**0.04** ^#^	7.3 (1.05–51.81)	**0.04** ^##^	5 (0.70–35.49)	0.108^###^
**Maternal regular opioid use (OM), Daughters**	0.57 (0.11–2.75)	0.48	0.49 (0.09–2.69)	0.42^#^	0.53 (0.09–3.11)	0.49^##^	0.5 (0.08–2.80)	0.431^###^
**Maternal smoking, Sons**	3.6 (0.59–21.93)	0.165	3.17 (0.48–20.77)	0.228^#^	3.30 (0.50–21.73)	0.214^##^	1.2 (0.12–11.86)	0.876^####^
**Maternal smoking, Daughters**	1.03 (0.14–7.52)	0.97	1.33 (0.16–10.62)	0.782^#^	1.02 (0.14–7.48)	0.981^##^	1.5 0.17–13.22)	0.715^####^

OR: odd ratio, CI: confidence interval.

Long RTL: relative telomere length higher>median.

*Adjusted for sex and Mother’s age.

**Adjusted for sex and Mother’s education.

***Adjusted for sex and Mother’s smoking.

**** Adjusted for sex and Mother’s regular opioid use (OM).

^#^Adjusted for Mother’s age.

^##^Adjusted for Mother’s education.

^###^ Adjusted for Mother’s smoking.

^####^Adjusted for Mother’s regular opioid use (OM).

On the other hand, the data does not support a significant association between female offspring’s leukocyte telomere length and maternal opioid use (Table 2).

Maternal smoking has been shown to affect neonate telomere length, although there is controversy in various reports in whether it leads to longer or shorter telomeres [25, 26, 46]. Here, we performed regression analysis to assess the association of maternal smoking and neonate elongated telomere. We did not find a significant association between maternal smoking and neonate telomere length in the crude and adjusted models with and without neonate sex stratification (Table 2). However, due to its importance in literature, we included this parameter as a confounder in our regression model assessing the relationship between maternal opioid use and neonate telomere length that abrogates p-value <0.05 or significancy of the association observed between maternal opioid use and neonate elongated leukocyte telomeres (Table 2). This indicates that the association we observe between maternal opioid use and long telomere in newborn leukocyte may be affected by smoking of mothers and it may act as a confounding parameter. It could also be because of our limited sample size.

Although low in statistical power, our results point to a potential connection between maternal opioid use and elongated leukocyte telomeres in male offspring only. Previous studies have shown an association between shorter leukocyte telomers in newborns and low birth weight [47–49]. Neonates born to mothers with opioid consumption in our study have significantly lower average birth weight (3140.92±488.91 in control vs. 2828.5±526.15 in OM groups, p-value = 0.02, please see Table 1 and Fig 1A for the ranges and distribution of birthweight). Despite lower birthweight in OM group, the male neonates in this group display higher odds ratio of longer telomers; therefore, we asked whether there is a suppressor mediating role for birth weight which reversed some of the effect of mother substance use on male newborn leukocytes telomere length. We categorized newborns to heavy and non-heavy categories based on the median birthweight value estimated for male and female neonates (2850 and 2925 grams, respectively) (Table 1). Comparing the odds ratio of logistic regression model with and without neonate categorical heavy/non-heavy variable as an intermediate factor, the results showed that when the effect of neonate heavy weight is controlled in the model, the odds ratio is significantly increased (Model1 OR: 5.5 vs. Model 2 OR:13.5, Table 3), which may indicate a suppressor mediating role for neonate low weight in OM group. It is important to note that due to our small sample size (after stratification for neonate sex), the power of the analysis is low. Future studies are warranted to further verify this observation. Nevertheless, based on the present dataset, we suggest that there is a positive correlation between mother opioid consumption and telomere length in male offspring which may have been indirectly suppressed by the effect of neonate low weight associated with mother opioid use (estimated mediation proportion = -177%, Table 3). On the other hand, controlling for neonate’s BMI as the potential mediator in the logistic regression model, we did not find a significant change in the odds ratio of long-telomere associated with mother opioid use (estimated mediation proportion = -2%, Table 3) (proportion mediation<10%).

**Table 3:** Mediation (suppressor) effect of neonate weight on the association between OM and long telomere.

	Model 1		Model 2*			Model 3**		
Long RTL	OR(95% CI)	p-value	OR(95% CI)	p-value	Mediation Proportion	OR(95% CI)	p-value	Mediation Proportion
Mother opioid use, Sons	5.5 (1.14–26.41)	0.033	13.5 (1.38–131.42)	0.025	-177%	5.5 (1.05–29.71)	0.043	-2%

% Mediation proportion: the degree to which neonate weight or BMI categorical parameter might mediate (suppresses) the effect of OM on neonate RTL.

*Intermediate parameter (heavy birth weight) added to the model.

**Intermediate parameter (high birth BMI) added to the model.

Long RTL: RTL longer than median.

Heavy birth weight: neonate weight higher than median.

OM: opioid using mother, RTL: relative telomere length, CI: confidence interval.

Overall, our analyses indicate a relationship between maternal opioid use and leukocyte telomere length in male offspring. Future studies with larger population sizes are required to further assess this observation.

## Discussion

Substance use has been linked to acceleration of normal aging by previous studies, indicating a role for accelerated telomere shortening [33–36]. Multiple studies have researched the features of abstinence syndrome, anthropometric measures and neurological characters of neonates exposed to prenatal opioid. However, research on the long-term health consequences affecting adulthood of offspring of opioid user parents are scarce. We previously found a dose-sensitive association between obesity in adulthood and parental opioid inhaling, suggesting that exposure to parental opioid may result in long-term health consequences in offspring [50]. Opioid use has been shown previously to induce aging in adults by shortening the telomere length, which promoted us to ask whether it may affect aging and aging biomarkers trans-generationally in the offspring of users? In the current study, we aimed to measure relative telomere length in the umbilical cord blood of newborns born to mothers with regular opioid consumption. Telomere length is known as an important marker of aging and its measurement in newborns is considered as a possible marker to predict the future health consequences of parental exposures. Surprisingly, we found a significant association of a long telomere in male neonate offspring with maternal opioid use. In daughters no significant association was observed between telomere length and maternal opioid use. Larger sampled studies are warranted in the future to verify this association and investigate the underlying mechanisms. Opioid misuse has been associated with shorter telomeres in adults [36, 37]. In contrast, we observed elongated telomeres in neonates born to mothers with regular opioid use. How does the effect differ in adults and neonates exposed to opioids? Our finding is consistent with some other reports that show a maternal life-time/long-term stress either did not lead to shorter neonatal telomeres or were associated with longer telomeres in neonates in contrast to gestational stresses (short-term) [28, 30, 51, 52]. The underlying suggested mechanism to explain this phenomenon is a compensatory higher induction of telomerase activity in embryos with mothers exposed to stress for a long-time, which results in elongated telomeres. This is suggested to occur as an adaptation programming in embryo to enhance their fitness upon stressful conditions. Why this compensatory mechanism does not work in adults themselves may be related to the fact that telomerase activity starts at early stages of embryonic development and is suppressed in most of tissues in adults [53]. Therefore, in adults, most of the cells do not have any telomerase activity and telomere maintenance. But in an embryo, most of the cells have active telomerase and telomere length protection. Thereby, one plausible explanation for our observation may be the higher induction of telomerase activity in neonates with mothers who use opioid regularly compared to normal control. Another explanation for our results may be related to the cytogenetic stress that opioid use may exert to cells. It is possible that a cytogenic damage to the progenitor cells of leukocytes imposed by opioid use led to halted cell cycle and replication, resulting in lower cell division and longer telomeres.

Whether opioid-induced RTL increase we observe in neonates born to opioid using mothers is beneficial or not? Answering this question requires follow-up studies that record health and clinical features of the participants in the future to see whether there is an association between their longer telomeres at birth with lower risk of chronic disease. Therefore, our study reports a change in telomere length and our data is not sufficient to make any conclusion regarding the effect on health of neonates. However, another point that needs to be considered is that the longer telomeres of leukocyte may in fact indicate a cytogenetic damage which has resulted in halted cell cycle and cell division and consequent longer telomeres. Future studies are warranted to check the lymphocytes regarding their cytogenetic damage and number to answer this question more thoroughly. Our study is a staring report as the first investigation of maternal opioid use in relation to telomere length of neonates with limited evidence to make any clinical conclusion.

Concurrent elongated telomers with cytogenetic damage to the leukocytes and lowered levels of cytokines has been reported in neonates prenatally exposed to cigarette smoke of mothers, in a study on 169 mother-neonate dyads by Almanzar et al., [25]. However, based on previous reports in the literature, no study has directly studied leukocyte count and cytogenetic status after prenatal opioid exposure in human subjects. In this regard, to the best of our knowledge there is only two reports, one assessing in vitro effect of opioid treatment on cultured whole cord blood cells of neonates, which observed a suppression of most of cytokines [54], which is more reflective of the effect of opioid administration in infants (post-natal) rather than the prenatal exposure to opioids. The other report studied rat animal model prenatally exposed to opioid and found elevated in vitro activation of peripheral blood mononuclear cells (PBMCs) and increased cytokine and chemokine production indicative of immune hyperactivity [55]. We suggest future studies that would directly assess cell count, cytokine measurement and cytogenetic damage levels in blood of human neonates born to opioid consumer mothers to provide a better answer to this question.

In our study, neonates of opioid using mothers have significantly lowered birth weight. Previous studies have linked low birthweight with shorter telomeres [47–49]. Adding neonates’ birthweight as the intermediate factor to our logistic regression analysis, we found a significant increase in odds ratio of elongated leukocyte telomeres associated with maternal opioid use in male offspring only, suggesting a suppressor mediation role for neonate weight in the association of newborn long telomers and maternal opioid use. However, the small sample size after stratification for neonate sex in our study, warrants future studies to verify this observation.

Maternal smoking has been shown to affect neonate telomere length, although contrasting reports have led to controversy whether it results in shorter or longer telomeres [25, 26, 46]. In our sample, running regression models, we did not find a significant association between maternal smoking and neonate telomere length. However, due to its importance in literature, we included this parameter as a confounder in our regression model assessing the relationship between maternal opioid use and neonate telomere length. Based on the regression models when mother opioid use is the exposure and long telomere is the dependent variable, adding mother’s smoking to the model abrogates p-value<0.05 or significancy. This indicates that the association we observe between maternal opioid use and long telomere in newborn leukocyte may be affected by smoking of mothers and it may act as a confounding parameter. It could also be because of our limited sample size. Future larger studies are required to perform stratified analysis by smoking and opioid use of mothers and address this question.

There is controversy on the effect of maternal smoking on neonate telomere length. For Example, the abovementioned study by Almanzar et al. found elongated telomeres in neonates born to smoking mothers [25]. In their study neonate weight was significantly lower in smoking mothers’ group and this factor was controlled in the regression models [25]. A contrasting study by Salihu et al., on a smaller sample size showed shorter telomeres in infants born to smoking mothers. However, this study did not include birthweight in their telomere analysis [38]. It is possible that smoking exerts cytogenetic stress to the neonate lymphocytes and results in longer telomeres as a direct effect, and indirectly results in shorter telomeres by lowering infants’ birthweight acting potentially through epigenetic alterations [56–59]. For example, as suggested by Antoun et al., DNA methylation alterations at Homeobox Telomere-Binding Protein 1 (HMBOX1) gene associated with lowered birthweight in neonates born to smoking mothers [56]. Regarding our results with opioid consumer mothers, future larger studies are required to measure telomere length as well as the level of replication of different lymphocytic cells and the level of cytogenetic damage to blood cells’ DNA, as well as, the epigenetic profile of genes which are important in telomere regulation and provide more conclusive evidences on this subject.

Our results displayed sex-differential telomere length in response to maternal opioid use only in male offspring, providing evidence of sex differences in the impact of maternal exposures on newborn telomere length. The reason for this phenomenon is not understood. Consistently, some previous tests which investigated various maternal risk/protective factors before and during gestation found similar distinction that only male offspring displayed a significant response to maternal stress or protective exposures such as smoking and mental illness [60–62]. Our findings are also consistent with other studies in adults that indicated stronger effect of stress on telomere shortening in males compared to females [63, 64]. The sex-differential fetal response to maternal exposures may be due to hormonal differences or may indicate X-linked mechanisms for determination of telomere length.

Limitations of the current study include the size of the sample, which may have affected the power of analysis. Specifically, in sensitivity analysis for sex, the small sample size has led to low statistical power to support the result. Another limitation of our study is that the drug use information is based on self-reports of individuals participating in the study. Previous studies which measured the bias in opioid use self-reporting by testing biological samples for residues of opioid indicated misreporting [65–69]. Also, some level of misclassification and recall biases are probable in self-reporting. Therefore, errors may occur in measurements leading to some deviations from reality (incidence of bias in estimates). Nevertheless, the level of this self-reporting bias varies due to factors such as sex, age, sociodemographic and geographical states defining the level of social stigma associated with substance use [67, 70–72]. Geographical and social parameters have led to lower social stigma of substance use in the city that the current study is undertaken (18), which provides more support toward the validity of our data on opioid use. Consistently, previous studies have provided supportive evidence for this notion indicating a high sensitivity rate of opium use in a Turkmen population in Iran which traditionally use opioid as a medicine with less associated social stigma [73].

Overall, our study indicated longer telomeres in blood cells of male newborns with opioid user mothers compared to normal controls. This unique finding warrants more validating studies in the future as this is the first report that indicates effect of maternal opioid use on offspring blood cells telomere.

## Supporting information

S1 Data Stata file including the anonymized basic values for the measured and collected information for each participant of the present study. (DTA)
